# Blood pressure, glycemic status and advanced liver fibrosis assessed by transient elastography in the general United States population

**DOI:** 10.1097/HJH.0000000000002835

**Published:** 2021-03-01

**Authors:** Stefano Ciardullo, Tommaso Monti, Guido Grassi, Giuseppe Mancia, Gianluca Perseghin

**Affiliations:** a Department of Medicine and Rehabilitation, Policlinico di Monza, Monza; b Department of Medicine and Surgery; c Department of Statistics and Quantitative Methods; d Clinica Medica, University of Milano Bicocca, Milan, Italy

**Keywords:** cirrhosis, diabetes, fibroscan, fibrosis, hypertension, nonalcoholic fatty liver disease

## Abstract

Few studies investigated the role of different predictors of advanced liver fibrosis in unselected populations. Here, we estimate the prevalence of steatosis and fibrosis in the general United States population by means of transient elastography and evaluate the impact of blood pressure (BP) and diabetes on disease severity.

**Methods:**

This is a cross-sectional study of United States adults participating in the 2017–2018 cycle of the National Health and Nutrition Examination Survey. Participants underwent a transient elastography examination, and liver steatosis and fibrosis were estimated through the controlled attenuation parameter (CAP) score and liver stiffness measurement (LSM), respectively.

**Results:**

Four thousand, three hundred and seventy-one participants had reliable transient elastography and BP readings. Steatosis (CAP ≥ 248 dB/m), advanced fibrosis (LSM ≥ 9.6 kPa) and cirrhosis (LSM ≥ 13 kPa) were present in 56.9, 5.5 and 2.9% of participants, respectively. After controlling for potential confounders, risk of steatosis increased proportionally going from participants with optimal (reference) to those with normal [odds ratio (OR) 1.24, 95% confidence interval (CI) 0.83–1.86], high normal (OR 1.41, 95% CI 1.01–1.97) and elevated BP (OR 1.64, 95% CI 1.21–2.21), whereas no significant association was found between BP status and liver fibrosis. Conversely, presence of diabetes increased the risk of both steatosis (OR 2.15, 95% CI 1.49–3.11) and advanced fibrosis (OR 2.25, 95% CI 1.36–3.72).

**Conclusion:**

Liver steatosis and fibrosis are highly prevalent in the multiethnic United States adult population, raising concerns for future incidence of cirrhosis and its complications. BP status was associated with a progressively higher risk of steatosis, whereas obesity and diabetes were consistently associated with both steatosis and fibrosis.

## INTRODUCTION

Due to the global obesity pandemic reaching unprecedented heights, with a prevalence of 42% in adults from the United States population [1], a series of cardiometabolic traits associated with visceral adiposity have risen accordingly. Among them, nonalcoholic fatty liver disease (NAFLD) became the most common form of chronic liver disease, affecting a quarter of the adult world population and growing disproportionately in children and adolescents [2,3]. Although the majority of patients with NAFLD do not develop liver-related events, given the prevalence in the general population, its more severe form, nonalcoholic steato-hepatitis (NASH), is the most rapidly growing indication for liver transplant, ranking second in the United States [4].

A large body of evidence suggests that advanced liver fibrosis is the strongest predictor of future development of clinically relevant liver disease, including decompensated cirrhosis, hepatocellular carcinoma and liver-related death [5]. Nonetheless, its prevalence in the general adult population is unknown, as liver biopsy, the gold standard technique for its assessment, is an invasive procedure not well suited for large population studies. Among available noninvasive techniques, transient elastography is one of the most promising and best validated and correlates strongly with histological stages of liver fibrosis [6]. Moreover, evidence suggests that two common comorbidities in patients with NAFLD, diabetes and hypertension, increase the risk of fibrosis progression [7–9].

Therefore, in the present study, we analyzed data from the 2017 to 2018 cycle of the National Health and Nutrition Examination Survey (NHANES) to evaluate the impact of blood pressure (BP) and diabetes on advanced fibrosis, as measured by transient elastography, in adults from the general United States population.

## MATERIALS AND METHODS

NHANES is a cross-sectional survey program conducted in the United States by the National Center for Health Statistics, part of the Centers for Disease Control and Prevention. In the survey, a stratified, multistage, clustered probability sampling design is applied with the aim of including individuals representative of the general, noninstitutionalized United States population of all ages. To obtain enough data on minorities, oversampling of non-Hispanic black, Hispanic and Asian persons, people with low income and older adults is performed. The survey starts with a structured interview conducted in the home, which is followed by a standardized health examination (including physical examination and laboratory tests) conducted at a mobile examination center. Full methodology of data collection is available elsewhere [10].

The Centers for Disease Control and Prevention Research Ethics Review Board approved the original survey, and all adult participants provided written informed consent. The present analysis was deemed exempt by the Institutional Review Board at our institution, as the dataset used in the analysis was completely de-identified.

### Clinical and laboratory data

Body measurements including height (cm), weight (kg) and waist circumference (cm) were ascertained at the mobile examination center using a standardized protocol; BMI was calculated as weight in kilograms divided by height in meters squared and obesity defined as a BMI at least 30 kg/m^2^.

Laboratory methods for measurements of hemoglobin A1c (HbA1c), plasma glucose, lipid profile, alanine aminotransferase (ALT), aspartate aminotransferase (AST), γ-glutamyltranspeptidase (GGT), platelet count, serum and urine creatinine and albumin are reported in detail elsewhere [11]. LDL cholesterol was calculated using the Friedewald formula [12].

Estimated glomerular filtration rate (eGFR) was computed according to the Chronic Kidney Disease Epidemiology Collaboration (CKD-EPI) equation [13], and CKD was defined as an eGFR less than 60 ml/min per 1.73 m^2^. On the basis of the measured urine albumin to creatinine ratio (UACR), participants were defined as having normo-albuminuria (UACR < 30 mg/g), micro-albuminuria (UACR between 30 and 300 mg/g) or macro-albuminuria (UACR≥300 mg/dl).

Information on smoking status, history of heart failure, coronary artery disease (CAD) and stroke were based on self-report. Cardiovascular disease (CVD) was defined as a composite of CAD and stroke/transient ischemic attacks.

Hepatitis C virus infection was indicated by presence of viral RNA and/or a confirmed antibody test and hepatitis B virus infection as a positive surface antigen test, as described [14]. Alcohol consumption was estimated based on self-reported data on the amount and frequency of alcohol use within the previous year. It was considered significant if more than 30 g/day for men and more than 20 g/day for women [15].

### Blood pressure and glycemic status

BP was measured by certified physicians according to a protocol that follows procedures developed by the American Heart Association. Survey participants are asked to seat quietly for 5 min, after which three consecutive auscultatory blood pressure readings (measured 30 s apart from the same arm) are obtained using a mercury sphygmomanometer. In case the physician is unable to obtain valid measurements, a fourth attempt is made. On the basis of information on arm circumference obtained during physical examination, an appropriate size cuff is applied. In the present analysis, the mean of the three measurements was taken as the representative value for both SBP and DBP.

Hypertension was defined as a SBP value at least 140 mmHg and/or a DBP value at least 90 mmHg or currently taking antihypertensive drugs, in accordance with the European Society of Cardiology/Hypertension guidelines [16]. The remaining participants were further categorized as having optimal (SBP <120 mmHg and DBP <80 mmHg), normal (SBP 120–129 mmHg and/or DBP 80–84 mmHg) and high normal blood pressure (SBP 130–139 mmHg and/or DBP 85–89 mmHg).

In accordance with guidelines from the American Diabetes Association, diabetes was diagnosed if any of the following conditions were met: a self-reported diagnosis of diabetes; use of anti-diabetic drugs; an HbA1c at least 6.5% (48 mmol/mol); a fasting plasma glucose at least 126 mg/dl and a random plasma glucose ≥ 200 mg/dl [17].

### Transient elastography

Liver stiffness measurement (LSM) by transient elastography and controlled attenuation parameter (CAP) were measured by NHANES technicians after a 2-day training program with an expert technician, using the FibroScan model 502 V2 Touch (Echosens, Paris, France) equipped with both M and XL probes. The M probe was used initially unless the machine indicated use of the XL probe. Inter-rater reliability between NHANES technicians and expert FibroScan technicians was 0.86 for LSM (mean difference 0.44 ± 1.3 kPa) and 0.94 for CAP (mean difference 4.5 ± 19.8 dB/m).

Patients were asked to fast for at least 3 h and were placed in a supine position with the right arm fully abducted and measurements were made on the right liver lobe through an intercostal space. Examinations were considered reliable if at least 10 valid LSM values were obtained after a fasting time of at least 3 h, with an interquartile range/median less than 30%.

Median CAP values at least 248, 268 and 280 dB/m were considered indicative of S1, S2 and S3 steatosis [18]. A median LSM at least 8 kPa was considered indicative of significant (≥F2) fibrosis [19], whereas values at least 9.6 kPa and 13 kPa were considered indicative of F3 (advanced fibrosis) and F4 (cirrhosis), respectively [6,20].

### Study population

**FIGURE 1:**
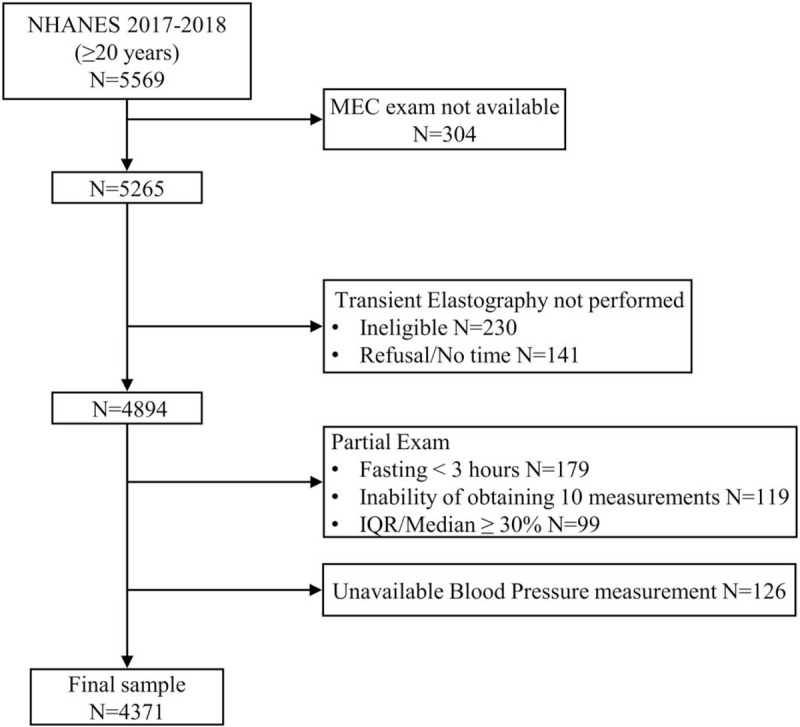
Flow-chart of the study participants. IQR, interquartile range; MEC, Mobile Examination Center; NHANES, National Health and Nutrition Examination Survey.

Five thousand, two hundred and sixty-five participants aged at least 20 years attended a mobile examination center visit. Among these, 230 participants were considered ineligible for transient elastography for different reasons (unable to lie down, currently pregnant, presence of an implanted electronic medical device, presence of lesions where measurements would be taken) and 141 additional patients were excluded because of refusal or insufficient time for the examination. Of the remaining 4894 patients, 397 had an incomplete examination because of fasting for less than 3 h (*n* = 179), inability of obtaining 10 valid measures (*n* = 119) and an IQR/median LSM value at least 30% (*n* = 99). After exclusion of 126 additional participants lacking blood pressure data, the final sample consisted in 4371 United States adults (Fig. 1).

### Statistical analysis

All analyses were conducted using SAS version 9.4 (SAS Institute Inc., Cary, North Carolina, USA), accounting for the complex survey design of NHANES. Appropriate weighting was used for each analysis, as suggested by the National Center for Health Statistics. Data are expressed as numbers and weighted proportions for categorical variables and as weighted means ± standard error (SE) for continuous variables.

Participants’ characteristics by BP status were compared using linear regression for continuous variables and the design-adjusted Rao--Scott chi-square test for categorical variables. Logistic regression analysis was performed in order to evaluate the effect of blood pressure and glycemic status on the presence of steatosis and fibrosis after adjustment for potential confounders.

A two-tailed value of *P* less than 0.05 was considered statistically significant.

## RESULTS

### Features of the study population

Characteristics of the study participants with optimal BP, normal BP, high-normal BP and hypertension are shown in Table 1. High normal BP and hypertension were highly prevalent in the general United States adult population, being present in 9.2 and 37.4% of participants, respectively. Mean age increased significantly from participants with optimal BP (38.4 ± 0.8 years) to patients with hypertension (59.9 ± 0.55 years, *P* < 0.001), as did the prevalence of male participants (38.6 and 53.1% in the same two groups, *P* < 0.001). Similarly, as BP status worsened, so did the cardiometabolic profile, as shown by the progressively increasing levels of BMI, waist circumference and liver function tests. This was also the case for the prevalence of most comorbidities including heart failure, chronic kidney disease and an altered UACR. In particular, the prevalence of CKD was 6.2% in the overall population and it progressively increased with higher BP, reaching 14.1% in patients with hypertension. The prevalence of diabetes mellitus was relatively low in participants with optimal BP (2.7%) and increased progressively in participants with normal (8.4%), high-normal (8.5%) and elevated (25.7%) BP (*P* trend < 0.001). On the other hand, no significant differences in the prevalence of both hepatitis B and hepatitis C positivity were found across BP groups, and no clear pattern was found with regards to cigarette smoke. Finally, the proportion of non-Hispanic black individuals increased progressively from optimal to elevated BP.

**TABLE 1:** Features of the study population according to blood pressure category

	Entire cohort (*n* = 4371)	Optimal (*n* = 1347; 37.3%)	Normal (*n* = 623; 16.1%)	High-normal (*n* = 411; 9.2%)	Hypertension (*n* = 1990; 37.4%)	*P* value
Age (years)	47.9 ± 0.57	38.4 ± 0.82	43.4 ± 1.12	47.0 ± 0.93	59.9 ± 0.55	<0.001
Men [*N* (%)]	2171 (49.0 ± 0.92)	537 (38.6 ± 1.51)	347 (54.1 ± 3.22)	249 (65.8 ± 3.90)	1038 (53.1 ± 1.38)	<0.001
Race-ethnicity [*N* (%)]						0.028
Non-Hispanic white	1493 (62.7 ± 2.58)	442 (60.7 ± 3.21)	218 (64.6 ± 2.61)	130 (59.4 ± 4.09)	703 (64.6 ± 3.03)	
Non-Hispanic black	1013 (11.2 ± 1.58)	358 (9.6 ± 1.37)	147 (8.8 ± 1.94)	104 (13.4 ± 2.38)	389 (13.3 ± 1.80)	
Non-Hispanic Asian	631 (5.6 ± 0.93)	248 (6.1 ± 1.02)	116 (5.2 ± 0.89)	98 (5.9 ± 1.25)	551 (5.4 ± 0.97)	
Hispanic	998 (15.6 ± 1.92)	228 (18.8 ± 2.66)	94 (15.3 ± 2.05)	60 (17.7 ± 2.84)	249 (12.0 ± 1.56)	
Other	236 (4.9 ± 0.63)	71 (4.8 ± 0.99)	48 (6.1 ± 1.03)	19 (3.6 ± 1.48)	98 (4.7 ± 0.74)	
BMI (kg/m^2^)	29.5 ± 0.27	27.4 ± 0.32	29.7 ± 0.57	30.8 ± 0.62	31.1 ± 0.28	<0.001
Waist circumference (cm)	100.1 ± 0.72	93.3 ± 0.79	99.8 ± 1.29	104.2 ± 1.61	106.2 ± 0.78	<0.001
Current smoke [*N* (%)]	791 (16.9 ± 1.23)	253 (17.6 ± 2.14)	133 (19.5 ± 2.30)	96 (24.4 ± 3.70)	309 (13.3 ± 1.15)	0.019
CVD [*N* (%)]	446 (7.6 ± 0.72)	24 (1.5 ± 0.27)	29 (3.3 ± 0.62)	23 (5.3 ± 1.74)	370 (16.2 ± 1.50)	<0.001
Heart failure [*N* (%)]	109 (1.6 ± 0.25)	6 (0.2 ± 0.14)	3 (0.18 ± 0.13)	2 (0.23 ± 0.17)	98 (4.0 ± 0.60)	<0.001
CKD [*N* (%)]	340 (6.2 ± 0.63)	17 (1.3 ± 0.40)	17 (1.6 ± 0.59)	10 (1.8 ± 0.98)	296 (14.1 ± 1.35)	<0.001
Liver enzymes (IU/l)						
ALT	23.3 ± 0.44	20.1 ± 0.41	26.0 ± 1.19	28.5 ± 2.46	24.4 ± 0.55	<0.001
AST	22.3 ± 0.30	20.5 ± 0.42	22.8 ± 0.68	25.3 ± 1.97	23.3 ± 0.41	<0.001
GGT	30.1 ± 0.63	23.3 ± 1.14	27.9 ± 1.55	42.7 ± 4.60	35.4 ± 0.99	<0.001
Albumin (g/dl)	4.1 ± 0.01	4.1 ± 0.02	4.1 ± 0.02	4.0 ± 0.02	4.0 ± 0.01	<0.001
Platelet count (10^9^/l)	244.7 ± 2.90	245.5 ± 3.57	252.1 ± 3.50	243.5 ± 3.91	240.0 ± 3.42	0.776
Diabetes mellitus [*N* (%)]	818 (12.8 ± 0.61)	59 (2.7 ± 0.52)	79 (8.4 ± 1.69)	49 (8.5 ± 2.34)	631 (25.7 ± 1.12)	<0.001
Hepatitis C virus [*N* (%)]	41 (1.0 ± 0.37)	7 (0.4 ± 0.25)	3 (0.3 ± 0.19)	6 (2.9 ± 2.31)	25 (1.5 ± 0.55)	0.240
Hepatitis B virus [*N* (%)]	25 (0.2 ± 0.06)	5 (0.1 ± 0.07)	4 (0.3 ± 0.21)	3 (0.19 ± 0.11)	13 (0.33 ± 0.13)	0.196
UACR (mg/g)						<0.001
<30 [*N* (%)]	3738 (90.2 ± 0.42)	1268 (94.9 ± 0.71)	569 (94.9 ± 0.86)	373 (94.6 ± 1.38)	1528 (82.1 ± 0.79)	
30–300 [*N* (%)]	482 (8.2 ± 0.48)	65 (4.8 ± 0.73)	39 (4.1 ± 0.83)	32 (5.0 ± 1.32)	346 (14.3 ± 0.89)	
> 300 [*N* (%)]	104 (1.6 ± 0.19)	5 (0.3 ± 0.18)	8 (1.0 ± 0.34)	4 (0.4 ± 0.19)	87 (3.6 ± 0.51)	

Data are expressed as number [weighted proportions ± standard error (SE)] for categorical variables and as weighted means ± SE for continuous variables. Linear regression and Rao--Scott chi-square test were used to compare groups. ALT, alanine aminotransferase; AST, aspartate aminotransferase; CKD, chronic kidney disease; CVD, cardiovascular disease; GGT, gamma-glutamyltranspeptidase; UACR, urinary albumin creatinine ratio.

### Prevalence of steatosis and fibrosis

**FIGURE 2:**
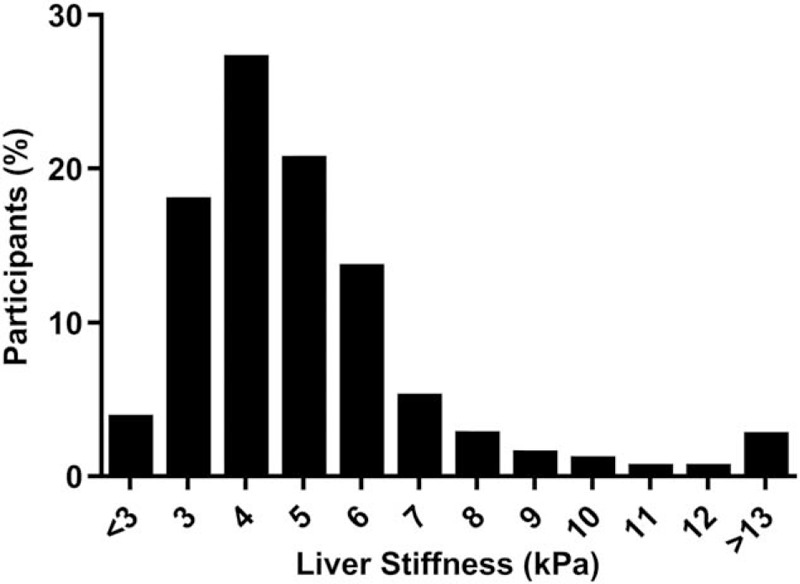
Distribution of liver stiffness measurement in the study population.

Prevalence of S1 and S3 steatosis in the overall population was 56.9% [95% confidence interval (CI) 54.1–59.8%] and 38.8% (95% CI 36.4–41.8%), respectively. In accordance with the worsening cardiometabolic profile, participants with higher BP readings had a higher prevalence of liver steatosis, estimated through CAP values. As shown in Table 2, S3 steatosis was present in 22.8% of participants with optimal BP values and increased in prevalence in those with normal (38.1%), high-normal (46.6%) and elevated (53.2%) BP (*P* trend < 0.001). Distribution of LSM values in the entire sample was skewed, as illustrated in Fig. 2. Weighted prevalence of significant fibrosis (LSM ≥ 8 kPa) in the overall population was 9.1% (95% CI 7.6–10.7%), advanced fibrosis (LSM > 9.5 kPa) was present in 5.5% of participants (95% CI 4.6–6.7%) and 2.9% (95% CI 2.2–3.8%) had elastographic evidence of cirrhosis (LSM≥13 kPa). Moreover, advanced liver fibrosis estimated by transient elastography was uncommon in participants in the optimal BP group (1.9%) but increased progressively with higher BP, reaching a prevalence of 8.5 and 8.8% in patients with high-normal and elevated BP (*P* trend < 0.001).

**TABLE 2:** Distribution of liver steatosis and fibrosis estimated through controlled attenuation parameter and liver stiffness measurement across blood pressure categories

	Entire population (*n* = 4371)	Optimal (*n* = 1347, 37.3%)	Normal (*n* = 623, 16.1%)	High-normal (*n* = 411, 9.2%)	Hypertension (*n* = 1990, 37.4%)
CAP (dB/m)					
<248 [*N* (%)]	1788 (43.1 ± 1.43)	783 (59.6 ± 1.78)	246 (44.4 ± 2.86)	155 (37.3 ± 3.63)	604 (27.5 ± 1.60)
248–268 [*N* (%)]	472 (10.2 ± 0.76)	137 (10.7 ± 1.09)	63 (10.4 ± 1.97)	34 (7.6 ± 2.09)	238 (10.1 ± 0.89)
268–280 [*N* (%)]	340 (7.9 ± 0.64)	97 (6.9 ± 0.88)	56 (7.1 ± 1.03)	29 (8.5 ± 2.49)	158 (9.2 ± 1.26)
>280 [*N* (%)]	1770 (38.8 ± 1.32)	330 (22.8 ± 1.32)	257 (38.1 ± 3.11)	193 (46.6 ± 3.44)	990 (53.2 ± 2.12)
LSM (kPa)					
<8 [*N* (%)]	3913 (90.9 ± 0.72)	1301 (96.9 ± 0.60)	564 (89.9 ± 1.05)	369 (85.7 ± 3.85)	1679 (86.9 ± 0.97)
8–9.5 [*N* (%)]	168 (3.6 ± 0.40)	19 (1.2 ± 0.33)	32 (5.5 ± 1.01)	17 (5.8 ± 1.97)	100 (4.3 ± 0.68)
9.6–13 [*N* (%)]	163 (2.6 ± 0.29)	15 (1.1 ± 0.34)	17 (2.4 ± 0.78)	12 (3.8 ± 1.74)	119 (4.0 ± 0.43)
>13 [*N* (%)]	127 (2.9 ± 0.37)	12 (0.8 ± 0.23)	10 (2.2 ± 0.540)	13 (4.7 ± 2.46)	92 (4.8 ± 0.67)

Data are expressed as number (weighted proportion ± standard error). CAP, controlled attenuation parameter; dB/m, decibel per meter; kPa, kilopascal; LSM, liver stiffness measurement.

### Independent predictors of steatosis and fibrosis

As cardiovascular risk factors and metabolic disease tend to cluster in the same patients, we performed multivariable logistic regression analysis to identify which factors are independently associated with steatosis and fibrosis. As shown in Table 3, male sex, non-Hispanic Asian and Hispanic ethnicity as well as higher age, BMI, ALT and GGT values were associated with the prevalence of any degree of steatosis (CAP ≥ 248 dB/m), whereas non-Hispanic black ethnicity was associated with a lower risk. In the same analysis, both diabetes and BP were significantly associated with steatosis, with a stepwise increase in risk going from normal BP to hypertension. Results were robust when a higher cut-off, indicative of S3 steatosis was used. A sensitivity analysis including only participants without viral hepatitis and significant alcohol consumption showed superimposable results (Supplementary Table 1).

**TABLE 3:** Multivariable logistic regression model assessing the contribution of several predictors on the odds of any or severe steatosis estimated through controlled attenuation parameter in the studied population

	CAP ≥248 dB/m			CAP ≥280 dB/m		
Characteristic	OR	95% CI	*P* value	OR	95% CI	*P* value
Sex						
Men	1.0			1.0		
Women	0.63	0.48–0.83	0.001	0.74	0.58–0.94	0.013
Race-ethnicity						
Non-Hispanic white	1.0			1.0		
Non-Hispanic black	0.47	0.37–0.60	<0.001	0.52	0.39–0.70	<0.001
Non-Hispanic Asian	1.97	1.54–2.53	<0.001	1.67	1.16–2.39	0.005
Hispanic	1.38	1.09–1.75	0.008	1.48	1.12–1.94	0.005
Blood pressure category						
Optimal	1.0			1.0		
Normal	1.05	0.75–1.48	0.777	1.24	0.83–1.86	0.303
High-normal	1.27	0.95–1.70	0.114	1.41	1.00–1.97	0.048
Hypertension	1.47	1.09–1.97	0.012	1.64	1.21–2.21	0.002
Age (per year)	1.03	1.02–1.03	<0.001	1.02	1.01–1.03	<0.001
BMI (kg/m^2^)	1.22	1.19–1.26	<0.001	1.19	1.17–1.21	<0.001
AST (IU/l)	0.99	0.98–1.01	0.367	0.98	0.97–0.99	<0.001
ALT (IU/l)	1.01	1.00–1.02	0.024	1.03	1.02–1.04	<0.001
GGT (IU/l)	1.01	1.00–1.01	0.033	1.00	1.00–1.01	0.023
Diabetes mellitus						
No	1.0			1.0		
Yes	2.12	1.43–3.13	0.002	2.15	1.49–3.11	<0.001

ALT, alanine aminotransferase; AST, aspartate aminotransferase; CAP, controlled attenuation parameter; CI, confidence interval; GGT, gamma-glutamyltranspeptidase; OR, odds ratio.

The same predictors, with the addition of serum albumin concentration and platelet count, were included in a multivariable logistic regression model with advanced fibrosis as dependent variable (Table 4). In this analysis, male sex, increasing age, higher AST and GGT values and presence of diabetes, but not BP status, were associated with the outcome. Moreover, similar results were found for probable cirrhosis, apart from a nonsignificant difference related to sex and a significant negative association with platelet count and non-Hispanic black ethnicity. Also in this case, inclusion of only participants without viral hepatitis and significant alcohol consumption did not significantly alter results (Supplementary Table 2).

**TABLE 4:** Multivariable logistic regression model assessing the contribution of several predictors on the odds of advanced fibrosis and cirrhosis estimated through liver stiffness measurement in the studied population

	LSM ≥9.6 kPa			LSM ≥13 kPa		
Characteristic	OR	95% CI	*P* value	OR	95% CI	*P* value
Sex						
Men	1.0			1.0		
Women	0.53	0.30–0.95	0.032	0.68	0.29–1.56	0.357
Race-ethnicity						
Non-Hispanic white	1.0			1.0		
Non-Hispanic black	0.81	0.53–1.25	0.340	0.32	0.14–0.72	0.006
Non-Hispanic Asian	1.36	0.63–2.94	0.434	1.02	0.39–2.67	0.975
Hispanic	0.96	0.56–1.64	0.878	0.70	0.31–1.62	0.405
Blood pressure category						
Optimal	1.0			1.0		
Normal	1.09	0.53–2.24	0.827	0.95	0.38–2.33	0.904
High-normal	1.27	0.54–2.97	0.584	1.35	0.53–3.41	0.532
Hypertension	0.94	0.50–1.77	0.847	0.99	0.42–2.36	0.997
Age (per year)	1.04	1.01–1.06	0.002	1.03	0.99–1.07	0.063
BMI (kg/m^2^)	1.18	1.15–1.21	<0.001	1.17	1.13–1.21	<0.001
AST (IU/l)	1.03	1.01–1.06	0.003	1.03	1.01–1.06	0.010
ALT (IU/l)	1.01	0.99–1.02	0.312	1.01	0.99–1.03	0.174
GGT (IU/l)	1.00	1.00–1.01	0.036	1.00	0.99–1.01	0.360
Diabetes mellitus						
No	1.0			1.0		
Yes	2.25	1.36–3.73	0.002	2.05	0.95–4.44	0.068
Albumin (g/dl)	0.85	0.48–1.52	0.584	0.47	0.17–1.26	0.133
Platelet count (10^9^/l)	0.99	0.99–1.00	0.206	0.99	0.98–0.99	<0.001

ALT, alanine aminotransferase; AST, aspartate aminotransferase; CI, confidence interval; GGT, gamma-glutamyltranspeptidase; OR, odds ratio.

## DISCUSSION

In the present study, involving more than 4000 participants of different ethnic background from the general United States adult population, we show that advanced fibrosis and cirrhosis, estimated through transient elastography, were present in 5.5 and 2.9% of participants, respectively. Moreover, male sex, higher age, BMI, AST and GGT levels and diabetes mellitus were independently and positively associated with advanced fibrosis, whereas no independent relationship was found with BP status after correcting for potential confounders. On the other hand, both BP status and diabetes mellitus were significantly associated with higher prevalence of liver steatosis.

Few community-based studies have assessed the prevalence of liver fibrosis in the general population and investigated potential predictors. In a French study involving 1358 participants older than 45 years attending a medical check-up, 7.5% had significant fibrosis (LSM ≥8 kPa) and 0.75% had cirrhosis (LSM ≥13 kPa). Independent predictors were higher age, BMI, ALT and GGT levels and diabetes mellitus, with hypertension reaching borderline statistical significance [odds ratio (OR) 1.7, 95% CI 0.9–3.1, *P* = 0.08] [21]. Similar findings were obtained in a subsequent analysis of the Rotterdam study involving 3041 participants older than 45 from Rotterdam, the Netherlands. In particular, prevalence of significant fibrosis and cirrhosis (using the same cut-offs) was 5.6 and 0.6%, respectively [22]. Although point estimates seem to be slightly higher in the present study, especially with regards to cirrhosis, it should be noted that mean age in the two previous studies was higher (57.7 and 66.0 years, respectively), but participants had lower BMI (26.4 and 27.3 kg/m^2^) and almost all were of Caucasian origin. Although these two studies did not report data on steatosis by Fibroscan, in a recent study from Italy, Petta *et al*. [23] applied this technology to simultaneously assess steatosis and fibrosis in 890 individuals aged 18–90 years recruited at a shopping mall in Palermo, Sicily. Steatosis (CAP ≥ 248 dB/m) was present in 47% of participants and advanced fibrosis (LSM ≥ 9.6 kPa) in 6.5% of patients with steatosis. As far as predictors were concerned, hypertension was an independent risk factor for steatosis and diabetes for advanced fibrosis. Although these results are in line with our estimates, the higher prevalence of steatosis in the NHANES cohort could be because of the inclusion of individuals with significant alcohol consumption, which were excluded from the Italian study. The high prevalence of overweight-obesity and diabetes in the United States population is also probably responsible for the relatively high prevalence of CKD (6.2%) and increased albuminuria (9.8%), which in turn are strictly associated with BP levels.

Although a large body of epidemiological evidence exists on the independent association between hypertension and liver steatosis, cause-and-effect relationships are difficult to ascertain in cross-sectional studies [24]. Cohort studies showed that on the one hand, increased BP values predict the development and progression of NAFLD, and on the other hand, the presence of NAFLD predicts the risk of incident hypertension, suggesting a bi-directional relationship between the two conditions [25]. Several mechanisms have been proposed to account for this association. Both NAFLD and hypertension are closely linked with insulin resistance, a condition that favors salt and liquid retention and ectopic fat deposition, as well as with low-grade inflammation. Moreover, some studies showed that patients with NAFLD have a reduction in nitric oxide production, leading to a vasoconstrictive state, and are more prone to oxidative stress [24]. Finally, in a recent meta-analysis of paired liver-biopsy studies, baseline hypertension doubled the odds of fibrosis progression [8].

The lack of a significant association between BP status and liver fibrosis in our population could be related to the cross-sectional nature of the study. In fact, because of hemodynamic changes related to advanced liver disease, patients with hypertension may become normotensive during the development of cirrhosis [26], thereby influencing association estimates. Conversely, diabetes is a well-known risk factor for the future development of advanced fibrosis and cirrhosis [27,28]. In addition, as fibrosis progresses, risk of incident diabetes increases, contributing to the cross-sectionally detected association between the two conditions [29]. Finally, we confirm the major role of obesity in the development of both fibrosis and cirrhosis.

The present study has several limitations that need to be addressed. First, we could not separate the contribution of insulin resistance, which plays an important role in the development of both NAFLD and diabetes. Second, there are no universal cut-off guidelines for CAP and LSM, and proposed threshold from previous studies were applied. Therefore, in the absence of biopsy data, we could only provide estimates of liver disease.

In summary, we show that liver steatosis and fibrosis are highly prevalent in the multiethnic United States adult population, raising concerns for future incidence of cirrhosis and its complications. Although BP status was associated with a progressively higher risk of having any and severe steatosis, it did not predict the presence of advanced fibrosis or cirrhosis. Moreover, after controlling for potential confounders, a history of diabetes was consistently associated with both steatosis and fibrosis.

## ACKNOWLEDGEMENTS

Author contributions: All authors made substantial contributions to the conception and design or acquisition, analysis and interpretation of data. All authors drafted the article or revised it critically for important intellectual content. All authors approved the final version of the manuscript to be published. G.P. is the guarantor of this work.

### Conflicts of interest

There are no conflicts of interest.

## Supplementary Material

Supplemental Digital Content

## Supplementary Material

Supplemental Digital Content
