# Supplementary material for: Blood pressure, glycemic status and advanced liver fibrosis assessed by transient elastography in the general United States population
Source: J Hypertens. 2021 Mar 1;39(8):1621–7. doi: 10.1097/HJH.0000000000002835 (PMC9904436; doi:10.1097/HJH.0000000000002835)
Supplement: Supplemental Digital Content [file jhype-39-1621-s002.docx]

**Supplementary Table 2** Multivariable logistic regression model assessing the contribution of several predictors on the odds of advanced fibrosis and cirrhosis estimated through Liver Stiffness Measurement (LSM), after exclusion of participants with viral hepatitis and significant alcohol consumption.

|  | **LSM ≥ 9.6 kPa** | | | **LSM ≥ 13 kPa** | | |
| --- | --- | --- | --- | --- | --- | --- |
| **Characteristic** | **OR** | **95% CI** | **p-value** | **OR** | **95% CI** | **p-value** |
| Sex |  |  |  |  |  |  |
| Men |  |  |  |  |  |  |
| Women | 0.50 | 0.26 - 0.95 | 0.04 | 0.66 | 0.25 - 1.78 | 0.39 |
| Race-ethnicity |  |  |  |  |  |  |
| Non-Hispanic white |  |  |  |  |  |  |
| Hispanic | 0.98 | 0.55 - 1.76 | 0.95 | 0.84 | 0.39 - 1.80 | 0.63 |
| Non-Hispanic Black | 0.65 | 0.37 - 1.16 | 0.13 | 0.23 | 0.09 - 0.60 | 0.01 |
| Non-Hispanic Asian | 1.25 | 0.50 - 3.09 | 0.61 | 0.74 | 0.24 - 2.30 | 0.58 |
| Blood pressure category |  |  |  |  |  |  |
| Optimal |  |  |  |  |  |  |
| Normal | 0.91 | 0.40 - 2.06 | 0.81 | 0.95 | 0.27 - 3.33 | 0.94 |
| High normal | 0.75 | 0.26 - 2.18 | 0.57 | 0.64 | 0.13 - 3.02 | 0.55 |
| Hypertension | 0.86 | 0.44 - 1.68 | 0.64 | 1.31 | 0.59 - 2.87 | 0.48 |
| Age (years) | 1.03 | 1.00 - 1.05 | 0.02 | 1.02 | 0.98 - 1.05 | 0.36 |
| BMI (kg/m^2^) | 1.19 | 1.16 - 1.22 | <0.01 | 1.18 | 1.13 - 1.23 | <0.01 |
| AST (IU/L) | 1.04 | 1.02 - 1.07 | <0.01 | 1.05 | 1.02 - 1.07 | <0.01 |
| ALT (IU/L) | 0.99 | 0.97 - 1.01 | 0.37 | 0.99 | 0.97 - 1.01 | 0.16 |
| GGT (IU/L) | 1.01 | 1.00 - 1.02 | 0.12 | 1.00 | 1.00 - 1.01 | 0.15 |
| Diabetes mellitus |  |  |  |  |  |  |
| No |  |  |  |  |  |  |
| Yes | 2.32 | 1.17 - 4.58 | 0.02 | 2.45 | 0.88 - 6.85 | 0.08 |
| Albumin (g/dL) | 1.09 | 0.55 - 2.15 | 0.80 | 0.72 | 0.22 - 2.32 | 0.56 |
| Platelet count (10^9^/L) | 1.00 | 0.99 - 1.00 | 0.64 | 0.99 | 0.99 - 1.00 | 0.05 |

Abbreviations: OR, Odds Ratio; CI, Confidence Interval; BMI, body mass index; ALT, alanine aminotransferase; AST, aspartate aminotransferase; GGT, gamma-glutamyltranspeptidase.
